# The importance of immune gene variability (MHC) in evolutionary ecology and conservation

**DOI:** 10.1186/1742-9994-2-16

**Published:** 2005-10-20

**Authors:** Simone Sommer

**Affiliations:** 1 Animal Ecology & Conservation, Biocentre Grindel, University of Hamburg, Martin-Luther-King-Platz 3, D-20146 Hamburg, Germany

## Abstract

Genetic studies have typically inferred the effects of human impact by documenting patterns of genetic differentiation and levels of genetic diversity among potentially isolated populations using selective neutral markers such as mitochondrial control region sequences, microsatellites or single nucleotide polymorphism (SNPs). However, evolutionary relevant and adaptive processes within and between populations can only be reflected by coding genes. In vertebrates, growing evidence suggests that genetic diversity is particularly important at the level of the major histocompatibility complex (MHC). MHC variants influence many important biological traits, including immune recognition, susceptibility to infectious and autoimmune diseases, individual odours, mating preferences, kin recognition, cooperation and pregnancy outcome. These diverse functions and characteristics place genes of the MHC among the best candidates for studies of mechanisms and significance of molecular adaptation in vertebrates. MHC variability is believed to be maintained by pathogen-driven selection, mediated either through heterozygote advantage or frequency-dependent selection. Up to now, most of our knowledge has derived from studies in humans or from model organisms under experimental, laboratory conditions. Empirical support for selective mechanisms in free-ranging animal populations in their natural environment is rare. In this review, I first introduce general information about the structure and function of MHC genes, as well as current hypotheses and concepts concerning the role of selection in the maintenance of MHC polymorphism. The evolutionary forces acting on the genetic diversity in coding and non-coding markers are compared. Then, I summarise empirical support for the functional importance of MHC variability in parasite resistance with emphasis on the evidence derived from free-ranging animal populations investigated in their natural habitat. Finally, I discuss the importance of adaptive genetic variability with respect to human impact and conservation, and implications for future studies.

## Introduction

Many natural populations are threatened not only by a dramatic reduction in total area of available habitat but also by increasing habitat fragmentation and degradation leading to declining population sizes and barriers to gene flow if exchange of individuals between subpopulations is restricted [1-3]. Small populations often suffer from reduction of genetic diversity due to genetic drift and inbreeding effects [4-6]. Negative effects such as increased rates of allelic loss, fixation of deleterious alleles and decreased average individual heterozygosity relative to the overall population were observed by both, theoretical and empirical studies [7,8]. The loss of genetic variation can lead to short-term reduction of fitness components such as survival, reproductive output, growth rates and to impaired ability to adapt to long-term changes in the environment [7,9-13]. An increasing number of studies indicates that host genetic diversity plays an important role in buffering populations against pathogens and widespread epidemics [6,14-20]. Study of the genetic effects of population fragmentation is therefore of central importance for conservation biology [21].

Genetic studies of wild animals often employ neutral markers such as mitochondrial d-loop DNA (mtDNA), microsatellites or single nucleotide polymorphism (SNPs) to estimate the amount of variation present in individuals and populations [22-24]. While these markers are very informative for phylogenetic reconstructions and population history (bottleneck effects), for molecular clocks, to examine dispersal patterns of individuals (gene flow) and to classify individuals by relatedness and paternity analyses [25-28], the variation at neutral loci cannot provide direct information on selective processes involving the interaction of individuals with their environment or on the capacity for future adaptive changes [29,30]. However, these are issues of particular relevance in evolutionary ecology and conservation [31,32]. In addition, recent research in a variety of taxa and situations has revealed that evolution often occurs on contemporary timescales, often within decades (summarised in [32]). In some cases, the time span between the separation of populations might even be too short to leave a signal at neutral loci so that differences between populations are only detectable at genes under selection [33], such as those of the highly variable major histocompatibility complex (MHC). Contrary to neutral markers, MHC variability reflects evolutionary relevant and adaptive processes within and between populations and is very suitable to investigate a wide range of open questions in evolutionary ecology and conservation. The comparison with neutral markers allows the construction of null hypotheses concerning the diversity at selectively relevant genes and conclusions on the relevance of MHC polymorphism. One might argue that many recent studies report that individual heterozygosity at apparently neutral microsatellite markers is correlated with key components of individual fitness such as survival [34], fecundity [35], disease resistance [14,36] and lifetime reproductive success [37]. However, null results are likely to be underrepresented in the literature because of publication bias in favour of significant correlations [38]. A recent review and meta-analysis of both published and unpublished studies of the association between neutral marker heterozygosity and traits or components of individual fitness reported that associations were common, yet typically weak [39]. A correlation between individual heterozygosity at neutral genetic markers and components of individual fitness can arise in different ways, with the effects of inbreeding depression due to a genome-wide reduction in genetic variability (including fitness-relevant loci) and linkage disequilibrium to loci under selection being the most likely explanations [[6,38,40], see also [41]].

In this review, I first introduce general information about the structure and function of MHC genes, as well as current hypotheses and concepts concerning the role of selection in the maintenance of MHC polymorphism. The evolutionary forces acting on the genetic diversity in coding and non-coding markers are compared. Then, I summarise empirical support for the functional importance of MHC variability in parasite resistance with emphasis on the evidence derived from free-ranging animal populations investigated in their natural habitat. Finally, I discuss the importance of adaptive genetic variability with respect to human impact and conservation, and implications for future studies.

## Major histocompatibility complex (MHC): structure, function and selection mechanisms

### Structure and function

The MHC consists of a group of closely linked genes that constitute the most important genetic component of the mammalian immune system [42]. The MHC encodes cell-surface glycoproteins that bind antigens derived from pathogens or parasites and present them to T-lymphocytes which trigger the appropriate immune response. Two major groups of MHC genes can be distinguished. MHC class I genes play an essential role in the immune defence against intracellular pathogens by binding peptides mainly derived from viral proteins and cancer infected cells. They are expressed on the surface of all nucleated somatic cells. In contrast, MHC class II genes are predominantly involved in monitoring the extracellular environment by presenting peptides mainly derived from parasites to the T-cells (e.g. bacteria, nematodes, cestodes) [43,44]. They are primarily expressed on antigen-presenting cells of the immune system, such as B cells and macrophages. Within class II genes, most research in mammals focuses on the second exon of DRB genes because these loci code for parts of the functionally important antigen binding sites (ABS) [45]. Alternatively, the β-chain in general is used if loci assignment is not possible due to missing information (e.g. in teleost, [46,47]). The class II region genes are closely linked in humans and all other mammals examined, and variants at these genes are generally in strong linkage equilibrium [48]. Thus, the pattern observed for DRB loci should be a good indicator of the genetic variation in other class II genes and even some other less closely linked genes in the MHC [49-51].

Genes within the MHC involved in antigen presentation constitute the most polymorphic loci known in vertebrates [52,53]. The variability of the MHC-molecules is correlated with the diversity of the T-lymphocyte receptors which in turn determine the disease and parasite resistance of an organism and thus may influence the long-term survival probability of populations [54-57]. The antigen binding sites show high levels of variation not only in the number of alleles but also in the extent of sequence variation between alleles [58]. Under neutrality theory, the rate of synonymous nucleotide substitution (d_s_) is predicted to be larger (d_s _> d_n_) than the rate of non-synonymous substitution (d_n_) because non-synonymous substitutions change the amino acid composition and are thereby likely to be deleterious [59,60]. However, several studies demonstrate that the ABS display more non-synonymous than synonymous substitutions (d_n _> d_s_) ([61,62], reviewed by [19]). This cannot be explained by a higher mutation rate in this specific region [58,61,62]. The emerging general view is that the determinant role in shaping patterns of nucleotide diversity in MHC genes is balancing selection [19,59,60,63]. Balancing selection results not only in the maintenance of large numbers of alleles in populations, but also in greatly enhanced persistence of allelic diversity over extremely long time periods relative to neutral genetic variation [64], an observation termed '*trans-species evolution of polymorphism*' [42]. The subsequent alteration in ABS allows binding of a diverse array of antigens [61,62,65].

### Selection mechanisms

Two main types of balancing selection ('*heterozygote advantage hypothesis*' and '*frequency-dependence selection*') have been suggested as important in retaining high levels of genetic diversity at the MHC in humans and vertebrates (reviewed by [19,64,66-68]).

In evaluating the evolutionary potential of '*heterozygote advantage*' mechanism [69] a clear distinction between '*dominance*' (heterozygote advantage in a broad sense) and '*overdominance*' (heterozygote superiority) is necessary. The term '*dominance*' refers to heterozygotes that are as resistant as the most resistant homozygote (if the allele A is associated with resistance, then the genotype AB is as resistant as AA (AB = AA)). In this case the heterozygote advantage could be due to masking of susceptible alleles. Whereas there is some support for this selection mechanism among experimental infection studies using mainly congenic mice it is clearly not sufficient to maintain high MHC variability [68,70,71]. '*Overdominance*' seems to be the more efficient '*heterozygote advantage*' mechanism promoting MHC diversity. In this case, heterozygotes are expected to have higher fitness than either parental homozygotes especially if confronted with multiple species or strains of pathogens or parasites (the genotype AB has a higher fitness than AA (AB>AA) and BB (AB>BB) [72]). The assumption is based on the theoretical background that heterozygous individuals should be able to detect and present a wider range of pathogen-driven antigens due to a larger number of different MHC molecules, hence increasing the relative fitness of MHC heterozygotes compared with homozygotes [60,73]. Thereby, two different '*overdominance*' models have been suggested: a) '*symmetric overdominance*' or '*symmetric balancing selection*' [74], whereby all heterozygotes derive a similar selective advantage to homozygotes (= all heterozygous are selectively equivalent), and b) '*divergent allele advantage*' [75]. In the later it is speculated that heterozygotes carrying more divergent allelic sequences have a selective advantage relative to individuals carrying relatively similar alleles by presenting a broader spectrum of antigens to the immune system. To the best of my knowledge, the '*divergent allele advantage*'-hypothesis has never been applied in infectious disease studies but to explain the persistence of highly divergent MHC alleles over millions of years [75,76]. Richman and colleagues [77] used a theoretical model to confirm Wakeland's contention that MHC alleles are more divergent than expected under a model of balanced genetic polymorphism assuming selective equivalence of different alleles (but see [78-80]). Application of this model to MHC class IIb gene sequence data of deer mice (*Peromyscus maniculatus*) provided more support for the '*divergent allele advantage*' model than for the '*symmetric overdominance*' model for the maintenance of MHC polymorphism [77]. Thereby it is important to note that the analysis are based on assumptions of the coalescent models in which no gene conversion is allowed and therefore conclusions should be taken with care if such a mutational process is suspected [19].

The second mechanism, '*frequency-dependent selection*', occurs when an allele or genotype is favoured at one frequency, but disadvantaged at another frequency [73,81,82]. Host-parasite dynamics are considered as an coevolutionary arms race. Pathogens adapt to infect the most common host genotype, leaving rare genotypes least infected [83]. If alleles are favoured when they are rare, but selected against when they are common, a balanced polymorphism results. Thus, the '*frequency-dependent selection*' hypothesis is also described as '*rare-allele advantage hypothesis*', '*Red Queen hypothesis*' or '*moving-target hypothesis*' [84-87]. The hypothesis assumes the following details. Rare (e.g. new) MHC alleles that are more resistant to parasites cause an advantage to the host, spread through the population and become common. This increases selection on parasites to evade recognition by these common alleles. As the parasite antigenicity changes, the relative fitness of the common host genotypes decreases and provides a selective advantage to other rare alleles. The time-lag nature of these antagonistic coevolutionary responses could lead to a cycling of fitness values of different alleles/genotypes in both hosts and pathogens, and result in the maintenance of high genetic diversity. As a consequence of these processes, pathogen-driven selection varies over time and may differ among habitats/environments within the range of a species, such that one host MHC-allele is favoured at a certain time in one environment and selected against in another. This should lead to varying spatiotemporal selection directions in space and time ('*diversifying selection in space and time*') [88,89,91]. So far, only one study investigated variation in MHC frequencies over time in a natural population to test the assumptions of the frequency-dependent model. Westerdahl and colleagues [92] compared the temporal changes in allele frequencies of 23 class I alleles and 23 neutral microsatellites of Great reed warblers (*Acrocephalus arundinaceus*) in nine consecutive cohorts. The MHC alleles showed on average slightly higher variation in temporal fluctuations compared to the microsatellite alleles. The frequency of two specific class I alleles varied more between cohorts than expected from random, whereas none of the neutral markers showed fluctuations exceeding the expectation from stochastic variation. The authors suggested that the variation in MHC allele frequencies between cohorts is not a result of demographic events, but rather an effect of selection favouring different MHC alleles in different years. However, Westerdahl and colleagues [92] did not include investigations of parasites or pathogens dynamics for explaining this pattern.

In addition, reproductive mechanisms such as disassortative mating and maternal-foetal interactions have been suggested as alternative or complementary mechanisms maintaining MHC diversity (summarised by [68,87,93-97]). MHC dissimilar mating preferences might act to increase offspring heterozygosity ('*good-genes as heterozygosity hypothesis*' [98]), to provide offspring with a moving target of MHC alleles as protection against pathogens which rapidly adopt to the parental genotypes ('*rare-allele advantage hypothesis*', '*Red Queen hypothesis*', '*moving-target hypotheses*' [73,81,82,87], to avoid inbreeding or genetic incompatibility ('*genetic compatibility hypothesis*' [99]) or to achieve an optimal MHC diversity in offspring with respect to parasite resistance ('*allele counting hypothesis*' [46,47] but see [91]).

The actual cue used in MHC-based mate choice is thought to be based on odour which allows to distinguish MHC-identity (summarised by [87,100-102]). Peptides/MHC complexes that are not retained at the cell surface but instead are released into the extracellular space might appear in the urine and other body secretions and be used for interindividual communication [103,104]. In mammals, the vomeronasal organ is essential in odour-based social recognition by detecting pheromones and other chemosignals that carry information about gender, sexual and social status, dominance hierarchies, and individualities, but it has been difficult to define the molecular nature of these chemosignals. Recent studies provided evidence that MHC class I peptides serve as chemosensory signals in the vomeronasal organ by which individual MHC genotype diversity can be used as a relatedness marker and may influence social behaviour [105].

These diverse functions and characteristics place genes of the MHC among the best candidates for studies of mechanisms and significance of molecular adaptation in vertebrates [19,52,93].

## Evolutionary forces acting on the genetic diversity in coding and non-coding markers

The maintenance of genetic variation in natural populations in neutral parts of the genome under the non-selective evolutionary forces such as genetic drift and inbreeding depend not only on the number of individuals constituting a population, but also on the particular life history, the dispersal patterns (gene flow) and the breeding system of the species under study [106,107]. In contrast, the ability of natural populations to maintain genetic variation in functional genes depends on the selection pressures involved. Balancing selection is thought to counteract the effects of genetic drift and to retard the rate of fixation of alleles [58].

### Evidence for selection maintaining high MHC diversity despite restricted variability in non-coding markers

There is increasing evidence for high MHC diversity due to balancing selection in species with otherwise restricted diversity in non-coding markers. For example, the San Nicolas Island fox (*Urocyon littoralis dickeyi*) is the most monomorphic sexually reproducing animal population yet reported with respect to variation in neutral genetic markers. No variation has been discovered in supposedly neutral hypervariable microsatellite loci and multilocus fingerprints, for which the probability of genetic identity is commonly <1 in several millions. Such low levels of variation imply lower resistance to pathogens, reduced fitness, and problems in distinguishing kin from non-kin. However, high MHC diversity is probably still maintained in this population by balancing selection. It is assumed that periodic selection has rescued genetic variation at the MHC and, potentially other fitness-related genes ([108] but see also [90]). Another example was found in Hawaiian honeycreepers (*Vestiaria coccinea*) [109]. Natural selection has maintained variation within the MHC while mitochondrial d-loop sequences and cytochrome b sequences were invariant and allozymes revealed low variability probably due to a genetic bottleneck. Moreover, in fragmented Malagasy gray mouse lemur (*Microcebus murinus*) populations, the number of DRB-alleles and the gene diversity were still high [110] but microsatellite and mitochondrial marker showed very low levels of polymorphism [111]. In the same study area, also the introduced black rat (*Rattus rattus*) revealed a similar pattern of genetic polymorphism: high levels of variability in the functional important MHC DRB marker [Sommer, unpublished data] in contrast to low mitochondrial d-loop variability (five haplotypes) [112].

These studies indicate that until a threshold level, genetic variation at the MHC might persist due to balancing selection despite low levels of variability shown by neutral markers. The results support the importance of balancing selection as a mechanism to maintain variation in natural populations and expose the difficulty of using neutral markers as surrogates for variation in fitness-related loci [108].

### Processes leading to low variability in both coding and non-coding markers

The maintenance of polymorphism within populations is dependent on the product of selection intensity, mutation rate and effective population size [58,113,114]. Under certain circumstances strength of selection acting on MHC loci can be insufficient to maintain variation in small or fragmented populations for a long period of time. The effects of balancing selection and genetic drift on the genetic diversity of coding MHC class II (DQA) variability, neutral mitochondrial control region and microsatellite marker were recently investigated in 14 island and two mainland populations of the Australian bush rat, *Rattus fuscipes *[115,116]. Both neutral marker sets revealed high levels of genetic variability over-all but clear signs of genetic drift such as little to no diversity in the small island populations and extreme differentiation between the populations. In the MHC, higher levels of heterozygosity were observed on two of the islands than would be expected under neutrality, but genetic drift played a dominant role in the majority of island populations leading to a decrease in the number of MHC alleles.

Similarly, historical events such as bottlenecks and founder effects but also constraints of the mating system can be reflected in low numbers of MHC alleles (for example in an Asian lion population (*Panthera leo persica*) [117]; cheetahs (*Aconyx jubatus*) [118]; Malagasy giant jumping rats (*Hypgeomys antimena*) [119-121]; Malagasy western forest mouse (*Macrotarsomys bastardi*) [120]; common hamsters in the Netherlands (*Cricetus cricetus*) [122]; Scandinavian beavers (*Castor fiber*) [123]; Swedish moose (*Alces alces*) [124,125]; musk ox (*Ovibos moschatus*) [126]; Spanish ibex (*Capra pyrenaica*) [127]; island population of desert bighorn sheep (*Ovis canadensis mexicana*) [128]; Arabian oryx (*Oryx leucoryx*) [129]; South African bontebok (*Damaliscus pygargus pygargus*) [130]; Przewalski's horses (*Equus przewalskii*) [55]; Northern elephant seals (*Mirounga angustirostris*) [131], fin whales (*Balaenoptera physlaus*] [132], sei whales (*Balaenoptera borealis*) [132], and black robins (*Petroica traversi*) [133]). Under these circumstances, the power of genetic drift has been stronger than the power of selection. As predicted by theoretical models [135], the reduced MHC polymorphism is usually correlated with low genome-wide genetic variation [89]. For example, cheetahs (*Aconyx jubatus*) show low MHC diversity, which correlates with a genome-wide loss of diversity presumably due to a genetic bottleneck about 10,000 years ago [118]. Also Northern elephant seals (*Mirounga angustirostris*) which were hunted near to extinction in the 19^th ^century lost most of the variability in allozymes, mitochondrial DNA, mini- and microsatellite loci and MHC class II loci [131,135].

## Empirical support for the functional importance of MHC variability in pathogen and parasite resistance

### Evidence for the functional importance of MHC variability and selective mechanisms derived from studies in humans or under experimental, laboratory conditions

While predictions of an association between MHC diversity and disease resistance are straightforward extensions of MHC theory, up to now, most of the empirical evidence has been derived from studies in humans or under experimental/laboratory conditions [19,67,136].

'*MHC heterozygote advantage*' [69] was indicated in humans by a slower progression to AIDS after HIV infection [137] and in a more effective clearance of hepatitis B viral infections [138]. In laboratory experiments, MHC-heterozygous mice showed reduced pathogenicity during bacterial and viral infection (streptococcus-induced lesions [139], *Salmonella*, *Lysteria *[70], *Salmonella enterica*, Theiler's virus [140]), an increased T-cell mediated immunity during lymphocytic choriomeningitis (LCM) infection [69] and they had a faster clearance rate of parasitic worms (*Heligmosomoides polygyrus *[141], *Schistosoma mansoni *[142]), than the average homozygote. Tumor incidence was lower and regression faster in heterozygous, rous sarcoma virus (RSV) infected chicken (*Gallus domestica *[143]). MHC class IIB heterozygotes had an increased survival rate in captive-raised fish, e.g. in Chinook salmon (*Oncorhynchus tsawytscha*) infected with a haematopoietic necrosis virus (HNV) [144] and in fluke-infected (*Gyrodactilus turnbulli*) Gila topminnows (*Poeciliopis o. occidentalis*) [54].

The '*frequency-dependent selection hypothesis*' [81,82] is engaged by both mathematical models [73,145] and some empirical studies that show correlations between certain alleles and disease resistance in humans (e.g. malaria [146], Epstein-Barr-virus [147], hepatitis B [148], leprosy, tuberculosis [67], *Heliobacter*-infected gastric cancer [149]). In humans, a correlation was observed between some MHC class II haplotypes and the clinical severity of cestode infections (*Echinococcus multilocularis*) [150]. Certain MHC alleles also played a role in resistance/susceptibility to a fungal disease (*Cryptococcus neoformans *[151]), infections with gastrointestinal nematodes in lab mice (*Trichinella spiralis *[152,153], *Nematospiroides dubius *[154], *Trichuris muris *[155]) and in straightbred Scottish Blackface sheep (*Ostertagia circumcincta *[156,157]). Associations between resistance and MHC genotype was found in chicken suffering from infection with Marek's disease (a tumour disease caused by a herpes virus [158]). Experimental evidence for MHC-allele-specific resistance to *Aeromonas salmonicida *bacteria [57,159] and to the infectious salmon anaemia virus (ISAV) was found in captive-raised Atlantic salmon (*Salmo salar *[160]).

### Evidence for the functional importance of MHC variability and selective mechanisms derived from studies in free-ranging animal populations in their natural environment

Whereas studies carried out under experimental or laboratory conditions can be better standardised to account for different parameters (e.g. in inbred congenic mice), they do not provide sufficient information to evaluate the ubiquity of pathogen-driven selective mechanisms acting in free-ranging animal populations in their natural habitat. Doing MHC research in wild vertebrates allows to test whether the results of studies on inbred congenic lab strains will hold in animals with a more diverse genetic background. Further, laboratory studies cannot reveal the effects of conditionally advantageous or deleterious alleles which will be discovered only in the presence of natural stress, such as spatially and temporally changes in climate, food availability, competition, and associated levels of parasitism [18,161]. Predicting the evolutionary potential of wild host populations in response to parasites requires at least a minimal understanding of the genetic basis for host resistance and heritability under field conditions, and the strength and mode of parasite-mediated selection [162]. Few studies have attempted to test for an association between MHC polymorphism and parasite resistance in wild populations under natural conditions [19]. Available information is summarised in Table 1.

**Table 1:** Evidence for pathogen-driven selection mechanisms in free-ranging vertebrate populations investigated in their natural environment.

**Host species**	**Host environment**	**Country**	**Infectious agent**	**Heterozygote advantage**	**Negative frequency-dependent selection**	**Reference**
Three-spined stickleback (*Gasterosteus aculeatus*)	Lakes and rivers	Germany	14 species of macroparasites	Supported in terms of a general diversity advantage; minimal parasitation at intermediate MHC class IIB diversity; population exposed to more diverse parasites had more different alleles.	Not investigated	[47]
Soay sheep (*Ovis aries*)	Large unmanaged population on an island	Scotland	Strongyle nematode	Not supported; heterozygosity is not the critical factor determining mortality in lambs and yearlings.	Common alleles (OLADRB 205, OLADRB 257) were associated with decreased lamb or yearling survivorship and a high incidence of parasitism; the rarer allele (OLADRB 263) with increased yearling survival.	[56]
Gray mouse lemur (*Microcebus murinus*)	Littoral rain forest	Madagascar	Seventeen nematode species; separate data analysis for (most common) single and multiple infections.	Not supported; heterozygosity was uncorrelated with infection status (being infected or not), the number of different nematodes per individual (NNI) as well as with the faecal egg counts (FEC, eggs/g faeces).	The common allele *Mimu*-DRB*1 was more frequently found in infected individuals, in individuals with high number of different nematode species infections (NNI) and faecal egg counts (FEC); the rare alleles *Mimu*-DRB*6 and 10 were more prevalent in not infected individuals and in individuals with low NNI and FEC values.	[174]
Yellow-necked mouse (*Apodemus flavicollis*)	Tree-dominated habitat	Germany	Eight nematode species; separate data analysis for (most common) single and multiple infections.	Not supported; heterozygosity did neither influence the infection status (being infected or not), nor the number of different nematode infections (NNI) nor the individual faecal egg count (FEC, eggs/g faeces) values.	Mice carrying allele *Apfl*-DRB*5 or the closely related allele *Apfl*-DRB*15 had an increased risk of being nematode infected and displayed higher FEC than individuals carrying other alleles; the allele Apfl-DRB*23 was associated with low FEC in separate analyses of the most common nematode.	[173]
Hairy-footed gerbil (*Gerbillurus paeba*)	Dunefield of the Southern Kalahari Desert	South Africa	Two different cestode species, six different nematode species	Not investigated	Gepa-DRB*15 was only found in not infected mice.	[172]
Striped mouse (*Rhabdomys pumilio*)	Dunefield of the Southern Kalahari Desert	South Africa	Eight different nematode species	Supported; heterozygosity did influence the infection status (being infected or not) and the individual faecal egg count (FEC) value with higher values observed in homozygous individuals.	The allele *Rhpu*-DRB*1 occurred more frequently in infected individuals and in individuals with high FEC values (high parasite load). In contrary, the allele *Rhpu*-DRB*8 occurred more often in individuals with low FEC values.	[163]

Under field conditions, associations between MHC heterozygosity and resistance/susceptibility to parasite infections have only been found in the African striped mouse (*Rhabdomys pumilio *[163]) and in three-spined sticklebacks (*Gasterosteus aculeatus *[47,164]) which seem to possess up to six MHC class II loci. In the later, a modification of a simple heterozygote advantage was identified as within individual fish, intermediate, rather than maximal allele numbers were associated with minimal parasite load [47,164]. This is explained by the fact that MHC-genes are involved in the preservation of T-cells during thymic selection. At some point, increasing the number of MHC molecules expressed should cause a net loss of T-cells and therefore negatively affect the organism [165] (but see also Borghans and colleagues [166] who used a simulation approach which revealed that several hundred alleles would be required to cause such a net loss of T-cells). Different allele numbers can be produced by both heterozygosity at single loci and differences in MHC class II gene duplication numbers across haplotypes [167]. At the moment it is not clear whether or not this selection pattern of intermediate, rather than maximal allele numbers is confined to species with a relatively flexible genomic architecture such as sticklebacks and other teleosts with haplotype variation in their MHC locus duplication numbers, or whether it represents a more general feature that has been overlooked in previous studies [168]. In mammals, a flexible MHC genomic architecture, namely the appearance of multiple MHC class II DRB loci with variable loci numbers between individuals has been described in rhesus macaques (*Macaca mulatta *[169]) and in California sea lions (*Zalophus californicus *[170]). The later possess up to eight different DRB loci in variable configurations among individuals but with low levels of allelic variation per loci. Preliminary evidence suggested an association between a certain MHC genotype and urogenital cancer. In contrary to sticklebacks, no relationship between the total number of unique DRB genes and the presence of cancer has been identified [171]. A possible relationship between the number of MHC alleles and parasitic load in mammals was also investigated in hairy-footed gerbils (*Gerbillurus paeba*) which possess two functional DRB loci [172]. Here, individuals carrying three different MHC alleles had significantly higher faecal egg count values than individuals with four alleles [172]. This is in accord with the theoretical background which assumes that animals containing more MHC alleles than others should be able to recognise a larger spectrum of pathogen-derived antigens and consequently be infected by less parasite species and/or to be generally less intensively infected [69].

An association between certain MHC alleles and disease resistance or susceptibility was found in a free-ranging sheep population (Soay sheep, *Ovis aries*) where MHC variants appear to play a major role in protection against strongyle nematode invasion, the most prevalent gastrointestinal parasite found [56]. As expected by the assumptions of the '*negative frequency-dependent selection*' ('*rare-allele advantage hypothesis*', '*Red Queen hypothesis*', '*moving-target hypotheses*') [73,81,82], the most common alleles OLADRB 205 and 257 (allele frequencies: 0.21–0.24) were associated with decreased lamb or yearling survivorship, whereas the rarer OLADRB 263 allele (allele frequency: 0.13) was associated with increased yearling survival (Table 1). Further evidence for the importance of certain MHC alleles and resistance or susceptibility to helminths was revealed in a common European rodent (yellow-necked mouse, *Apodemus flavicollis *[173]), in the two African rodent species mentioned before (*Gerbillurus paeba *[172], *Rhabdomys pumilio *[163]), and in a primate species (gray mouse lemur, *Microcebus murinus *[174]) (Table 1). Also in *R. pumilio*, it was the most common allele *Rhpu*-DRB*1 (allele frequency: 0.22) which occurred more frequently in infected individuals and in individuals with high faecal egg count values (indicating high parasite load) whereas the rare allele *Rhpu*-DRB*8 (allele frequency: 0.05) occurred more often in individuals with low FEC values (indicating low parasite load). Also in *M. murinus*, the common allele *Mimu*-DRB*1 (allele frequency: 0.33) was more frequently found in infected individuals and in individuals with a high number of different nematode species infections and faecal egg count values (eggs/g faeces) (indicating high parasite load), the rarer alleles *Mimu*-DRB*6 and *10 (allele frequencies: 0.11 and 0.06) were more prevalent in not infected individuals, in individuals with low number of different nematode species infections and faecal egg count values (indicating low parasite load). These examples demonstrate the frequency-dependence of selection between parasites and hosts in the form of a rare allele advantage in the host population.

### Evaluating the relative importance of balancing selective mechanisms

Right now there is still much debate whether '*heterozygote advantage*' or '*frequency dependent selection hypothesis*' is most important for balancing selection [89]. Most studies investigating '*heterozygote advantage*' compared the infectious disease outcomes of heterozygotes at a given MHC loci, as a group, to the outcomes of homozygotes at the same locus, as a group ('*population heterozygote advantage*' [70,175], examples see above) probably always due to restrictions in sample size. However, comparing the average performance of all heterozygotes against homozygotes, instead of using allele specific tests for '*overdominance*', can not distinguish whether the observed advantage is due to '*dominance*' or '*overdominance*'. Grouping all homozygotes and all heterozygotes, respectively, circumvent tests of the original hypothesis namely the superiority of heterozygotes over either corresponding homozygote [69] (see paragraph '*Selection mechanisms*' above) as this hypothesis is conditional on the alleles involved (and should be more precisely termed '*allele-specific overdominance*' [175]). However, a theoretical model showed that under a very wide range of assumptions about the relationship between homozygote and heterozygote infectious risk, '*allele-specific overdominance*' might be consistent with '*population heterozygote advantage*', e.g. a '*population heterozygote advantage*' might occur when the diversity of resistant alleles is sufficiently high and the diversity of susceptible alleles is sufficiently low [175]. But also the opposite might be true. Because of confounding effects of differences in frequencies of susceptible or resistant alleles, population level tests can, in a worse case, find a heterozygote advantage even when every heterozygote is at greater infection risk than either corresponding homozygote in allele-specific analyses [175]. Direct estimates of the allele-specific effects of heterozygosity relative to the corresponding homozygotes are rare. The most convincing experimental evidence for heterozygote advantage through '*allele-specific overdominance*' derived from McClelland and colleagues [140] using co-infections with multiple pathogens in MHC-congenic mice with reciprocal resistance/susceptibility profiles (but the authors did not test for fitness consequences). In humans, the only studies that directly compare the outcomes of heterozygotes to those of homozygotes for the same alleles derived from investigations of autoimmune but not from infectious diseases (summarised by [70,175]).

As mentioned above allele-specific analyses were most often impossible due to restrictions in sample size. In humans, recently a new approach to circumvent this problem was proposed by classifying alleles to supertypes based on shared binding motifs [176,177]. Though it is clear that the highly polymorphic HLA genes play a crucial role in the immune response, their great diversity is a major obstacle in distinguishing HLA allele-specific effects and complicates the attribution of specific alleles with the outcome of diseases. Collecting samples of the size needed for definitive results is often not feasible. The biological relevance of a classification scheme based on functional binding specificities is supported by a growing body of evidence of cross-presentation of specific peptide-binding motifs by different HLA molecules. Trachtenberg and colleagues [176] investigated the usefulness of grouping HLA alleles to supertypes by their overlapping peptide binding specificities in explaining the association between HLA polymorphism and HIV disease progression. Their study indicated that HLA-supertypes are highly predictive of viral load. Consistent with the rare-allele advantage model the authors could show the advantage of a rare HLA supertype in progression of HIV [176]. After more intense studies of the implications and limits of supertypes in large human data sets, this classification approach of common functional traits may also provide tools for the MHC research in natural endangered populations, where high allelic diversity causes problems in obtaining sufficiently large statistical sample sizes. Caution must be taken not to miss the effects of new, rare alleles by clustering alleles in functional types.

As outlined before, two different '*overdominance*' models have been suggested: a) '*symmetric overdominance*' or '*symmetric balancing selection*' [74], whereby all heterozygotes derive a similar selective advantage to homozygotes (= all heterozygous are selectively equivalent), and b) '*divergent allele advantage*' [75]. As almost all heterozygote advantage studies were carried out on the population level, so far, no effort has been made to differentiate between these two '*overdominance*' models in infectious disease studies. However, the '*divergent allele advantage*' hypothesis has recently been considered in MHC-dependent mate choice studies [121,178]. In the African striped mouse (*Rhabdomys pumilio*) where associations between MHC heterozygosity and resistance/susceptibility to parasite infections have been found [163], no significant correlation between pairwise genetic distances of heterozygotes (a measurement for allelic divergence) and infection rates (faecal egg count: log-transformed number of eggs per gram faeces) was found (Froeschke & Sommer, unpublished data). Another point that need to be mentioned is that only a few studies in natural populations indicating correlative evidence for '*heterozygote advantage*' combined MHC research with estimates of genome-wide diversity by using neutral markers and thus could rule out possible effects of genome-wide heterosis [47,137].

With respect to the '*frequency dependent selection hypothesis*', evidence for pathogen-resistant/susceptible alleles/haplotypes is not equally available. So far, more alleles/haplotypes were found to be associated with susceptibility to disease [67] (but see Table 1). This bias could be simply due to over presentation of human studies, in which the emphasis has been on finding disease-allele associations [179]. But it is also in line with theoretical predictions of host-parasite coevolution. Susceptibility is expected to be more common, because fast evolution of the parasite is assumed to fuel the arms race between them and their hosts. For most pathogens it is valid to assume a higher evolutionary potential compared to that of the host, because generation times are usually much shorter or effective population sizes of pathogen populations are larger [18]. The human HLA-A11 allele, for example, confers resistance to infection with Epstein-Barr-virus only in populations where the allele is rare. In populations with high frequency of this allele, virus strains have fixed a mutation that prevents presentation of immunodominant epitopes by HLA-A11 molecules [147]. Also certain HLA alleles are associated with a slower progression of HIV if they are rare and have no advantage if they are common (summarised by [176]). This shows that a fast-evolving pathogen is able to adapt to host defence.

Evaluating the relative importance of both balancing selective mechanisms, so far, more evidence is available for the importance of specific MHC-alleles in parasite resistance or susceptibility. It is conceivable that a rare allele may have a high fitness and at the same time a constant advantage for heterozygotes. Both hypotheses may be in accord with each other and are not mutually exclusive. But as most studies deal with single viral, bacterial or parasitic agents it was suggested that studies combining two or more pathogens may increase the amount of evidence for heterozygote advantage [57,136,140] (but see [173,174]). De Boer and colleagues [180] studied the degree of MHC polymorphism arising when '*heterozygote advantage*' is the only selection pressure by using mathematical models. The simulations revealed that '*heterozygote advantage*' on its own is not sufficient to explain the high population diversity of the MHC. This would require that the fitness contributions of all alleles would be unrealistically similar. '*Heterozygote advantage*' in pathogen resistance could, however, promote mating preferences for MHC-dissimilarity, which in turn drive high allelic diversity [52,68,70]. This could explain why MHC-heterozygous males have attributes important during sexual selection such as an increased antler development and body mass in white deer [181] and sexually attractive odour in stickleback fish [46]. In contrast, a study on sexual selection in pheasants (*Phasianus colchicus*) found that females prefer males with larger spurs, and that this sexually selected trait is associated with a particular MHC allele [182,183]. The overall view is emerging that although '*heterozygote advantage*' is clearly an important selection pressure additional frequency-dependent selection pressure is required. A theoretical model by Hedrick [89] indicated that the selective force from pathogens, which vary in space and time ('*diversifying selection in space and time*'), could maintain the genetic polymorphism in MHC genes. Since evolving pathogens mainly evade presentation by the most common MHC alleles in the host population, they provide a selective pressure for a large variety of rare alleles. Host-parasite coevolution would be sufficient to explain the large degree of MHC polymorphism [145].

In ongoing studies assessing the evolutionary genetic details of vertebrate host-parasite relationships and evidence for frequency-dependent parasite-driven selection four patterns ought to be evident: (1) parasitism reduces host fitness, (2) MHC alleles differ in susceptibility, (3) alleles frequencies change according to (2), and (4) in the longer term dynamics should encompass frequency-dependent allele frequency fluctuations [17]. Whereas (1) and (2) indicate the potential for selection and have been shown in recent studies in wildlife populations (e.g. [184], this review), so far evidence for (3) and (4) is limited. Ongoing investigations of the parasite-driven selection mechanisms under natural conditions should focus on temporal variation of pathogens, host fitness attributes and allele frequencies to test whether allele frequencies change accordingly in a cycling pattern.

### Functional differences of amino acid variation in the antigen binding sites

There is increasing evidence that pathogen escape from MHC-dependent immune system recognition may involve changes in only a few amino acids so that small binding-motif differences can lead to large differences in protection. Common mechanisms include changes in pathogen antigens (epitopes) that prevent binding (1) to the MHC-encoded cell surface glycoprotein or (2) to the T-cell receptor. (3) A third mechanism is molecular mimicry of host proteins that prevent T-cell receptor binding (T-cells that recognise host proteins are destroyed during thymic selection). For instance, a one-amino-acid difference in the antigen-binding region of the DRB*1302 allele abrogates its protection to malaria (summarised by [185,186]). In Malagasy mouse lemurs (*Microcebus murinus*), MHC-alleles associated with gastrointestinal nematode susceptibility (*Mimu*-DRB*1, *6 and *10) have unique amino acid motifs in the antigen binding sites (ABS) [174]. *Mimu*-DRB*1 associated with high parasite load differs from all other alleles by three unique amino acids, all of them located within the functional important ABS (aspartic acid in position 70, glutamic acid in position 71, lysine in position 74). Two of these ABS are mutated in *Mimu*-DRB*6 and *10 currently associated with low parasite load: the allele *Mimu*-DRB*6 has a unique motif at position 74 (glycine) and *Mimu*-DRB*10 at position 71 (methionine). In addition, only *Mimu*-DRB*6 and *10 possess the amino acid arginine located next to the ABS in position 78 [174] (position numbers after [62]). This indicates the functional differences of certain amino acids in the ABS and thus the influence of different amino acid compositions on parasite resistance.

So far, the molecular details of the interactions between helminth parasites and the intestinal components of the immune system are not as well understood as for viral or bacterial infections. However, huge progress was made in understanding the cellular and molecular mechanisms in the immune regulation by gastrointestinal helminth parasites in recent years. The recognition of gastrointestinal parasites and their antigens, and the initiation of the immune response occur in specialised lymph nodes in the epithelium of the gut wall, the so called Peyer's patches. In these Peyer's patches all cell types necessary for antigen presentation to CD4+ T-cells including MHC class II molecules are present. This activates a range of interacting processes against the parasite culminating in an inflammatory reaction in the intestinal mucosa and different effector mechanisms against the invading parasite (summarised in [186-191]).

## Importance of MHC variability in conservation

### Importance of adaptive genetic variability with respect to human impact

Human impact (e.g. habitat fragmentation, degradation, isolation, urbanisation, pollution) has diverse impacts on the ecology and genetics of both, vertebrate and parasite populations. It often causes a loss of genetic variation leading to short-term reduction of fitness components, and to an impaired ability to adapt to changing environments which in turn influences evolutionary outcomes [5,6,12,18,192]. Habitat degradation and climatic conditions are also crucial parameters in terms of distribution, transmission and developmental success of parasites and pathogens [18,192,193]. Such changes may have significant implications for outbreak patterns of pest species, the conservation of rare mammal species and their ecological functions, as well as associated veterinary and medical consequences for wildlife, lifestock and humans [194]. Rapid evolution (on the order of decades or shorter) has been supported by numerous examples from host-parasite systems, and it is now clear that pathogens can cause major shifts in the genetic composition of their hosts on short timescales [18,162,195]. Detectable changes in allele frequencies can occur between subsequent generations and can be a sensitive indicator for demographic changes in some species [196].

The effects of pollution on the MHC was investigated in the estuary killifish (*Fundulus heteroclitus*) [33]. Populations experiencing strong differences in antigenic challenges (PCB-contaminated versus unpolluted site) show significant differences in amino acid substitution patterns in a highly variable MHC class II B locus. However, whether MHC population profile differences represent direct effects of chemical toxicants or parasite-mediated selection need to be investigated [33]. The only study including an environmental variable such as habitat fragmentation in the analysis of associations of MHC-constitution and parasite burden was carried out in a subdivided mouse lemur population [174]. The work indicated that variation in MHC-allele frequencies in the fragments were linked to parasite load as certain alleles which differed in a few amino acids in the ABS from other alleles (see above) were associated to parasite resistance or susceptibility. Female mouse lemurs inhabiting the fragment with the highest parasite load had a lower fat deposition in the tail (important during the dry season) and therefore lower survival rates than populations of the three other fragments. In addition, the population size declined dramatically in recent years [197]. However, to clearly separate whether the higher parasite load in the respective fragment is due to the MHC-constitution of individuals inhabiting this fragment or due to other ecological factors associated with fragment size or degradation needs further investigations [174]. Nevertheless, the study suggests that the MHC-constitution might influence the long-term survival of small fragmented animal populations and indicates the functional importance of maintenance of MHC variability in declining or fragmented animal populations.

More studies in free ranging animal populations with respect to human impact are needed to allow more general conclusions on the importance of adaptive genetic variability in conservation. According to the theoretical background, temporal and spatial variation in the parasitic fauna will cause shifts of selective advantage of certain MHC-alleles in different areas changing over time. This should result in habitat- and climate-specific amino acid substitution patterns in the functional important ABS in relation to local pathogen-driven selective pressures. So far, empirical evidence for '*diversifying selection in space and time*' is limited. Studies on the interaction between environmental conditions and the expression of genetic covariation (the so called genotype-environment interaction) might be an important avenue for future work. Genotype-environment interactions have commonly been found in live history traits when multiple environments were considered reflecting the fact that genes influencing a trait in one environment may not be important in a different one [198]. In this context, host and parasite movement among habitat fragments could be crucial to both parasite persistence, and the spread and maintenance of resistance alleles and thus to allow ongoing coevolutionary processes. The role of metapopulation dynamics in maintaining the diversity of host resistance genes can be a matter of concern in conservation genetics aiming at the preservation of both current patterns and ongoing processes. As contemporary evolution is influenced by complex interactions among population size, genetic variation, strength of selection, and gene flow, the overall goal in conservation genetics – maintenance of short-term local adaptations and preservation of long-term adaptive potential – might be a challenging task [32].

### Relevance of MHC polymorphism for individual fitness and long-term persistence

**Figure 1:**
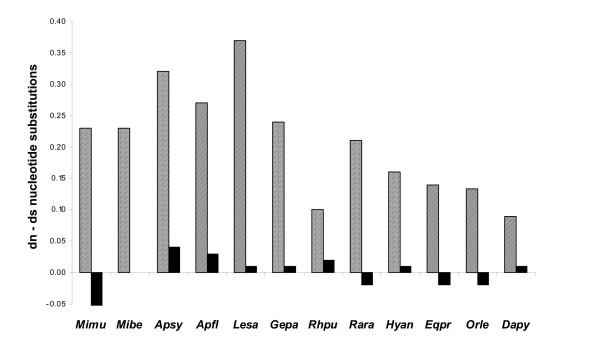
Differences between the rates of non-synonymous (d_n_) and synonymous (d_s_) substitutions (= d_n_-d_s_, amino acid sequence-changing substitution rate) in antigen-binding sites (ABS, shaded bars) and nonantigen-binding sites (nonABS, black bars) of MHC class II DRB exon 2 alleles of the species included in Table 2. Abbreviations follow the MHC nomenclature [42], the first two letters of the genus and the species name are used.

Genetic variation at MHC loci is thought to be important for resistance against pathogens, thereby increasing individual fitness and thus the long-term survival of endangered species [60,73]. Several studies have reported decreased pathogen resistance among MHC homozygotes, or an increase in pathogen susceptibility in inbred individuals in general. However, a direct link between pathogen-mediated population decline and low MHC variation has been difficult to demonstrate in natural populations [49]. Recent studies indicated that although MHC allele numbers are low in many bottlenecked species most of them still indicate a high degree of divergence between alleles. Table 2 summarises the number of functional important MHC class II DRB exon 2 alleles and sequence diversity in some free-ranging vertebrate populations investigated in their natural habitat. The comparison indicates that also species with a low number of different MHC alleles, such as the critically endangered Malagasy Giant Jumping Rat (*Hypogeomys antimena*, 5 alleles) whose geographic range was recently restricted to less than 20,000 ha, still have high levels of nucleotide and amino acid divergence between MHC DRB-alleles while mitochondrial d-loop sequences revealed very low variability [199-201]. A similar picture was revealed in the Przewalski's horse (*Equus przewalskii*, 6 alleles [55]), in the Arabian oryx (*Oryx leucoryx*, 3 alleles [129]) and in the South African bontebok (*Damaliscus pygargus pygargus*, 6 alleles [130]) (Table 2). Considering the nonABS, the ratio between non-synonymous (d_n_) and synonymous (d_s_) substitutions was significantly smaller (d_n _< d_s_) than unity in some species (Fig. 1) which indicates purifying selection acting on these codons depending on their respective function [202]. In contrary, comparisons of non-synonymous (d_n_) and synonymous (d_s_) substitution rates in parts coding for the functional important ABS revealed a significantly higher rate of substitutions (d_n _> d_s_) which change the amino acid constitution in the ABS and thus increase the divergence between alleles in all species irrespective of the number of MHC alleles still present (Fig. 1).

**Table 2:** Number of MHC class II DRB exon 2 alleles (ca. 200 bp) and sequence diversity in free-ranging vertebrate populations investigated in their natural environment. In addition, for comparison of variability levels of species with a low number of MHC alleles two studies of captive-bred populations are included. * DRB-locus is duplicated. N = sample size.

**Species**	**Order**	**Country**	**Nr of alleles (N)**	**Nr (%) of variable nucleotide positions**	**Nr (%) of variable amino acid positions**	**Nr (%) of amino acid differences between alleles**	**References**
*Microcebus murinus*	primates	Madagascar	14 (228)	71 (41.5)	31 (54.4)	5 (8.8) – 25 (43.9)	[110,174]
*Microcebus berthae*	primates	Madagascar	9 (42)	46 (26.9)	24 (42.1)	3 (5.3) – 19 (33.3)	Sommer et al., unpublished data
*Apodemus sylvaticus*	rodentia	Germany	38 (119)	71 (32.7)	38 (52.8)	2 (2.7) – 28 (38.8)	[206]
*Apodemus flavicollis*	rodentia	Germany	27 (146)	49 (22.6)	28 (38.9)	1 (1.4) – 21 (29.2)	[173]
*Leopoldamys sabanus*	rodentia	Borneo	28* (49)	85 (49.7)	39 (68.4)	4 (7.0) – 25 (43.9)	Lenz et al., unpublished data
*Gerbillurus paeba*	rodentia	South Africa	34* (40)	68 (39.8)	33 (57.9)	1 (1.8) – 19 (33.3)	[172]
*Rhabdomys pumilio*	rodentia	South Africa	20 (58)	43 (25.1)	23 (40.4)	1 (1.8) – 14 (24.6)	[163]
*Rattus rattus*	rodentia	Madagascar	13 (58)	72 (40.7)	33 (55.9)	3 (5.1) – 26 (44.1)	Sommer, unpublished data
*Hypogeomys antimena*	rodentia	Madagascar	5 (229)	37 (17.1)	19 (26.4)	6 (8.3) – 21 (29.2)	[201]
*Equus przewalskii*	perissodactyla	captive-bred	6* (14)	52 (20.8)	29 (34.9)	1 (1.2) – 22 (26.5)	[55]
*Oryx leucoryx*	artiodactyla	captive-bred	3 (57)	35 (14.8)	21 (26.9)	13 (16.7) – 17 (21.8)	[129]
*Damaliscus pygargus pygargus*	artiodactyla	South Africa	6 (45)	21 (8.4)	14 (16.9)	1 (1.2) – 13 (15.7)	[130]

The studies indicate that selection processes are able to maintain MHC polymorphism also under unfavourable conditions at least for a certain time which in turn might suggest that levels of variability in species with low numbers of MHC alleles might be sufficient to prevent immediate pathogen-mediated population decline. However, in such populations adaptive processes to changing conditions might be limited. An intriguing question is still what happens if new pathogens arise which differ from commonly encountered diseases by the respective populations. It is reasonable to assume that the maintenance and even more renewal of variation in functional important parts of the MHC such as in the antigen binding sites, either from mutation, recombination or immigration from other populations is an important genetic component in the cascade leading to an appropriate immune response, when combating new or coevolving virulent pathogens. It was suggested that the extremely low genetic variability in cheetahs (*Aconyx jubatus*) as a consequence of inbreeding depression due to a substantial bottleneck about 10,000 ago limits adaptation processes to temporary pathogens which might explain the high disease susceptibility nowadays [118,203]. As in other mainly endangered species, the proposed association cannot be tested as there are no genetically variable outbred cheetah population to compare with [21]. Samples from preserved bones or from museum specimens in the case of recently presumed bottlenecks would be necessary to directly assess historical levels of MHC variation and to evaluate the relative role of demographic changes in determining existing levels of genetic diversity at the MHC [127].

Concerning the role of MHC in conservation genetics, the potential functional role of background genes (non-MHC genes) in disease resistance should not be ignored. Human studies indicate that background genes might also play an important role in determining pathogen and parasite resistance, either by themselves or in a epistatic manner with MHC-genes (summarised by [179,204]. Many of the regulatory genes show high polymorphism and, for example, variation in the tumor necrosis factor gene promotor, cytokines such as interleucin receptor, γ-interferon receptor vitamin and D receptor has been associated to infectious diseases (summarised by [187,191,204]. Although evidence is accumulating that the MHC is one of the main factors controlling resistance to diseases [176,205] conservation genetics should focus on the preservation of both, MHC and genome-wide diversity. Thereby, how much MHC diversity is required to ensure long-term population viability remains a fundamental question in conservation genetics [133] and can only be investigated close follow-ups of the genetic and health status of declining populations.

## Conclusion

The diverse functions and characteristics place genes of the MHC among the best candidates for studies of mechanisms and significance of molecular adaptation in vertebrates. In contrary to neutral markers, MHC variability reflects evolutionary relevant and adaptive processes within and between populations and is very suitable to investigate a wide range of open questions in evolutionary ecology and conservation.

The selective effects from different pathogens appear to be the major driving force in the maintenance of MHC variation. Evidence of balancing selection at MHC genes has been found at different temporal scales. Selection in the distant past has been documented as an excess of nonsynonymous to synonymous substitutions, and as trans-species polymorphism. Selection in the recent past has been determined by excess heterozygosity compared to neutral theory expectations, differences in F_ST_-values compared to neutral theory, or excess linkage equilibrium. Selection in the current generation has been identified by measuring deviations from Hardy-Weinberg or random mating proportions, survival differences between homozygotes and heterozygotes, and correlations of disease resistance with MHC-allele or genotype [90]. Changes in certain amino acids in the functional important MHC-coded antigen binding sites and thus the amino acid compositions influence functional differences in pathogen and parasite resistance. It is reasonable to assume that the maintenance and even more renewal of variation in functional important parts of the MHC such as in the antigen binding sites, either from mutation, recombination or immigration from other populations is an important genetic component in the cascade leading to an appropriate immune response, when combating new or coevolving virulent pathogens and might be important in conservation genetics. Studies indicate the functional importance of MHC variability in pathogen and parasite resistance not only in humans or in model organisms under experimental, laboratory conditions where most of our current knowledge derived from, but also in wild animal populations investigated in their natural environment. Only field studies in free-ranging animal populations can reveal the effects of conditionally advantageous or deleterious alleles in the presence of natural stress (e.g. spatially and temporally changes in climate, food availability, and competition), associated levels of parasitism, and thus the ubiquity of pathogen-driven selective mechanisms and the importance of MHC diversity across taxa. The combination with an experimental approach under standardized laboratory conditions is needed to prove the causal relationships behind correlations observed in the field.

Right now it is not quite clear whether '*heterozygote advantage*' or '*frequency-dependent selection hypothesis*' is most important for balancing selection. Most studies investigating '*heterozygote advantage*' compared the infectious disease outcomes of heterozygotes at a given MHC loci, as a group, to the outcomes of homozygotes at the same locus, as a group ('*population heterozygote advantage*'). Comparing the average performance of all heterozygotes against homozygotes, instead of using allele specific tests for '*overdominance*' (= '*allele-specific overdominance*') circumvent tests of the original hypothesis namely the superiority of heterozygotes over either corresponding homozygote as this hypothesis is conditional on the alleles involved. Allele-specific analyses were most often impossible due to restrictions in sample size. In humans, recently a new approach to circumvent this problem was proposed by classifying alleles to supertypes based on shared binding motifs [176,177]. After more intense studies of the implications and limits of supertypes in large human data sets, this classification approach of common functional traits may also provide tools for the MHC research in natural endangered populations, where high allelic diversity causes problems in obtaining sufficiently large statistical sample sizes. Caution must be taken not to miss the effects of new, rare alleles by clustering alleles in functional types. So far, more evidence is available for the importance of specific MHC-alleles in parasite resistance or susceptibility. It is conceivable that a rare allele may have a high fitness and at the same time a constant advantage for heterozygotes thus both modes of balancing selection may act synergistically to enhance the maintenance of polymorphism.

Ongoing investigations of the parasite-driven selection mechanisms under natural conditions should focus on temporal variation of pathogens, host fitness attributes and allele frequencies to test whether allele frequencies change accordingly in a cycling pattern. Assessing the immunogenetic status of a population relative to another experiencing different suites of antigenic challenges will help to increase our knowledge on the importance of adaptive genetic variability in free ranging animal populations with respect to human impact and the role of the MHC in evolutionary ecology and conservation.
